# New anthropometric indices or old ones: which perform better in estimating cardiovascular risks in Chinese adults

**DOI:** 10.1186/s12872-018-0754-z

**Published:** 2018-01-30

**Authors:** Fei Wang, Yintao Chen, Ye Chang, Guozhe Sun, Yingxian Sun

**Affiliations:** 1 Department of Cardiology, Jinhua Municipal Central Hospital, 351 Mingyue Street, Wucheng District, Jinhua, 321000 People’s Republic of China; 2 Department of Cardiology, the First Affiliated Hospital of China Medical University, 155 Nanjing North Street, Heping District, Shenyang, 110001 People’s Republic of China

**Keywords:** CHD risk, Anthropometric parameters, WHtR, ABSI, BRI

## Abstract

**Background:**

Various anthropometric indices can be used to estimate obesity, and it is important to determine which one is the best in predicting the risk of coronary heart disease (CHD) and to define the optimal cut-off point for the best index.

**Methods:**

This cross-sectional study investigated a consecutive sample of 11,247 adults, who had lived in rural areas of China and were older than 35 years of age. Eight obesity indices, including the body mass index (BMI), waist circumference (WC), waist-to-hip ratio (WHR), waist-to-height ratio (WHtR), abdominal volume index (AVI), body adiposity index (BAI), body roundness index (BRI) and a body shape index (ABSI) were investigated. The risk of CHD was evaluated by the 10-year coronary event risk (Framingham risk score). Receiver operating characteristic (ROC) curve analyses were used to evaluate the predictive ability of the obesity indices for CHD risk.

**Results:**

Of the whole population, 3636 (32.32%) participants had a risk score higher than 10%. Those who suffered medium or high CHD risk were more likely to have higher mean anthropometric indices, except for BMI in males. In the multivariate-adjusted logistic regression, all these anthropometric measurements were statistically associated with CHD risk in males. After adjusting for all the possible confounders, these anthropometric measurements, except for ABSI, remained as independent indicators of CHD risk in females. According to the ROC analyses, ABSI provided the largest area under the curve (AUC) value in males, and BMI showed the lowest AUC value, with AUC varying from 0.52 to 0.60. WHtR and BRI provided the largest AUC value in female, and similarly, BMI showed the lowest AUC value, with AUC varying from 0.59 to 0.70. The optimal cut-off values were as follows: WHtR (females: 0.54), BRI (females: 4.21), and ABSI (males: 0.078).

**Conclusions:**

ABSI was the best anthropometric index for estimating CHD risk in males, and WHtR and BRI were the best indicators in females. Males should maintain an ABSI of less than 0.078, and females should maintain a WHtR of less than 0.54 or a BRI of less than 4.21.

**Electronic supplementary material:**

The online version of this article (10.1186/s12872-018-0754-z) contains supplementary material, which is available to authorized users.

## Background

Over the past several decades, cardiovascular diseases, which primarily consist of arteriosclerotic cardiovascular disease (ASCVD), have become an overall global burden [1]. The prevalence of cardiovascular disease primarily originates from the alarming rise of cardiovascular risk factors. Excess of adiposity, particularly visceral adiposity, is just one such risk factor [2–4].

In recent years, there has been increased understanding of the role that obesity plays in cardiovascular diseases. However, no anthropometric parameter has been continually better than others in the discrimination of cardiovascular diseases [5]. BMI is the most widely accepted index of adiposity. However, BMI is affected by age, gender, and ethnicity [6], and it cannot differentiate visceral adiposity and overall adiposity. Although WC takes abdominal obesity into account [7], it ignores height. Some studies have proposed WHtR, which does consider height, as the best anthropometric parameter for predicting cardiometabolic risk [8]. However, it is not always the best parameter. Some studies have shown that WC was more associated with CHD risk factors than WHtR in Caucasians [9].

Recently, some new anthropometric parameters have been proposed. The abdominal volume index (AVI) is calculated using waist circumference and hip, and one study has shown that it was a good anthropometric tool for estimating overall abdominal volume [10]. The body adiposity index (BAI) is a composite index that is based on hip circumference and height. It could differentiate visceral adiposity and overall adiposity, and it was considered to be a better index of body adiposity [11]. However, some studies have shown that BAI was not always precise in measuring adiposity [12]. BRI is another composite index that is based on WC and height. One study has shown that BRI could determine the presence of CVD [13]. A body shape index (ABSI) is calculated using waist circumference, BMI, and height. Some studies have shown that ABSI was closely associated with diabetes and hypertension [14, 15]. However, Maessen et al. have shown that ABSI was not a suitable index to identify CVD and CVD risk factors in the Netherlands [13]. Determining the optimal anthropometric indices for specific purpose and population has been challenging.

As few studies have considered these new anthropometric indices, it was unknown whether they could better identify CHD risk than the old indices. This study sought to determine which anthropometric parameter was the best predictor of CHD risk among rural populations in northeast China and to define the optimal cut-off point for the best anthropometric index, which can be used by doctors or health officials to assess CHD risk, subsequently reducing the CHD risk in patients by targeting these parameters to reduce their values.

## Methods

### Study population

This large-scale cross-sectional study was conducted in Liaoning Province from January 2012 to August 2013. A total of 11,247 adults who had lived in rural areas of Liaoning Province and were older than 35 years of age were selected through multistage stratified random cluster sampling. A total of 14,016 participants enrolled in this survey, and the response rate was 85.3%. We have excluded the participants who were pregnant or had missing data. Finally, a sample size of 11,247 was accepted.

### Data collection and measurement

Data were collected by professionals using a standard questionnaire administered through face-to-face interview. An additional file shows this in more detail [see Additional file 1]. Before the survey was performed, all investigators were invited to attend a training session that covered the purpose of this study, how to administer the questionnaire, the standard method of measurement, the importance of standardization, and the study procedures. After completing the training session, a test was used to evaluated the performance of the investigators. Only those who scored perfectly could become eligible investigators. During data collection, all investigators could receive further instructions and support.

The questionnaire included sociodemographic variables, smoking status and health history. Standard blood pressure was measured three times at 2-min intervals after at least 5 min of rest using a standardized automatic electronic sphygmomanometer (HEM-907; Omron). Weight, height, WC and hip circumference were measured according to a standardized protocol and technique, with the participants wearing light clothes and no shoes. Each item was measured twice and if the measures differed by more than 0.5 cm or 0.5 kg, respectively, a third measurement was taken. The average of the two closest measurements was used in the analysis. After at least 10 h of fasting, blood glucose and serum lipid levels were measured before breakfast using an Olympus AU640 AutoAnalyzer (Olympus, Kobe, Japan).

### Definitions

The Framingham risk score, which was used to predicted coronary event risk within 10 years, was calculated as per the ATP-III guidelines [16]. According to the tertiles of calculated 10-year FRS, the participants were classified as low-, intermediate-, or high-risk. In this study, the medium-risk and high-risk groups were combined as the “medium or high risk” group.

- BAI was calculated, as proposed by Bergman et al. [11].
- The ABSI was calculated using the formula described by Krakauer et al. [17].
- The BRI was calculated using the formula described by Wilson et al. [13].
- AVI was calculated using the following formula: AVI = [2 × (waist)^2^ + 0.7 cm (waist–hip)^2^]/1000.
- Hypertension was defined as a systolic blood pressure (SBP) ≥140 mmHg, and/or diastolic blood pressure (DBP) ≥90 mmHg, according to the JNC-7 report guidelines [18].
- Diabetes mellitus was diagnosed using the WHO criteria, FPG ≥7 mmol/L (126 mg/dl) and/or being treated for diabetes [19].
- Dyslipidaemia was defined as using lipid-lowering drugs or having one or more of the following measurements: TG ≥1.7 mmol/L, TC ≥5.2 mmol/L, HDL-C < 1.0 mmol/L and LDL-C ≥ 3.4 mmol/L [20].

### Statistical analysis

Data were calculated as the means and standard deviations (SD) (continuous variables) or as numbers and percentages (categorical variables). Student’s t-test was used to compare continuous anthropometric parameters and metabolic risks. The χ^2^-test was used to compare categorized anthropometric measurement and metabolic risks. Univariate logestic regression was conducted to explore the association between anthropometric parameters and CHD risk, while multivariate logestic regression adjust the variables of age, hypertension, diabetes, dyslipidaemia, current smoking, current drinking, education and physical activity to further explore their relationship. ROC analyses were used to compare the predictive ability and to determine the optimal cut-off values of the anthropometric parameters. The optimal cut-off value was the highest Youden index value (SEN + SPE - 1). The parameter with the largest area under the curve was accepted as the best indicator. All analyses were calculated using SPSS version 19.0 software, and a *P* value of < 0.05 was considered to be statistically significant.

## Results

Table 1 shows the characteristics of the participants. A total of 11,247 participants were included in this study. Males were older and had higher blood pressure levels and WC, height, WHR, and AVI measurements; however, the levels of other indices, such as TC, LDL, WHtR, BAI, and BRI, were significantly higher in females than in males. The prevalence of hypertension, previous myocardial infarction, FRS ≥ 10% and smoking were significantly higher in men than in women. However, the prevalence of overweight or obesity was higher in females.

**Table 1:** Characteristics of the study population: Chinese men and women aged 35 years and over, 2012–2013

Variable	Men (*n* = 5192)	Women (*n* = 6055)	*p*
	Mean (SD)	Mean (SD)	
Age(years)	54.00 (10.37)	53.11 (10.01)	< 0.001
Systolic blood pressure (mmHg)	143.38 (22.56)	139.77 (23.87)	< 0.001
Diastolic blood pressure (mmHg)	83.78 (11.81)	80.53 (11.49)	< 0.001
TC(mmol/L)	5.17 (1.04)	5.29 (1.12)	< 0.001
TG(mmol/L)	1.66 (1.34)	1.62 (1.34)	0.11
LDL(mmol/L)	2.88 (0.79)	2.97 (0.84)	< 0.001
HDL(mmol/L)	1.41 (0.42)	1.41 (0.34)	0.78
BMI(kg/m^2^)	24.74 (3.54)	24.87 (3.76)	0.06
Height (cm)	166.48 (6.29)	155.68 (6.05)	< 0.001
WC (cm)	83.75 (9.72)	81.23 (9.66)	< 0.001
WHR	0.87 (0.08)	0.85 (0.08)	< 0.001
WHtR	0.50 (0.06)	0.52 (0.06)	< 0.001
AVI	14.22 (3.32)	13.39 (3.26)	< 0.001
BAI	26.85 (3.63)	31.23 (4.30)	< 0.001
BRI	3.48 (1.11)	3.87 (1.29)	< 0.001
ABSI	0.077 (0.005)	0.077 (0.006)	0.58
FRS ≥10%	3132 (60.32%)	504 (8.32%)	< 0.001
current smokers	57.28% (2974)	16.48% (998)	< 0.001
Hypertension^a^	53.44% (2775)	48.20% (2919)	< 0.001
Overweight or obesity (BMI ≥25 kg/m^2^)	43.86% (2277)	46.01% (2786)	< 0.05
Diabetes^b^	9.84% (511)	10.80% (654)	0.1
Previous myocardial infarction	1.31% (68)	0.96% (58)	< 0.05
Dyslipidaemia^c^	37.17% (1930)	35.67% (2160)	0.1

Data are expressed as mean (SD) or %(n)

*SD* standard deviation, *TC* total cholesterol, *TG* triglycerides, *LDL* low density lipoprotein, *HDL* high density lipoprotein, *BMI* body mass index, *WC* waist circumference, *WHR* waist:hip ratio, *WHtR* waist:height ratio, *AVI* abdominal Volume Index, *BAI* body adiposity index, *BRI* body roundness index, *ABSI* a body shape index, *FRS* Framingham risk score

^a^Hypertension was defined as a systolic blood pressure (SBP) ≥140 mmHg, and/or diastolic blood pressure (DBP) ≥90 mmHg, according to the JNC-7 report guidelines

^b^Diabetes was diagnosed using the WHO criteria, FPG ≥7 mmol/L (126 mg/dl) and/or being treated for diabetes

^c^Dyslipidaemia was defined as using lipid-lowering drugs or having one or more of the following measurements: TG ≥1.7 mmol/L, TC ≥5.2 mmol/L, HDL-C < 1.0 mmol/L and LDL-C ≥ 3.4 mmol/L

Student’s t-test and χ2-test

Table 2 shows the relationship between the anthropometric indices and CHD risk. Females who suffered medium or high CHD risk had significantly higher mean anthropometric measurements. Similarly, these anthropometric measurements, except for BMI, were significantly higher in males with medium or high CHD risk.

**Table 2:** Association of anthropometric parameters with CHD risk^a^ in Chinese adults: men and women aged 35 years and over, 2012–2013

	Men					Women				
	Low CHD risk		Medium or high CHD risk			Low CHD risk		Medium or high CHD risk		
	(*n* = 2060)		(*n* = 3132)			(*n* = 5551)		(*n* = 504)		
Index	Mean	SD	Mean	SD	*p*	Mean	SD	Mean	SD	*p*
BMI^a^	24.67	3.67	24.78	3.45	0.29	24.78	3.74	25.90	3.82	< 0.001
WC^a^	82.85	9.80	84.34	9.63	< 0.001	80.79	9.55	86.00	9.61	< 0.001
WHR^a^	0.86	0.07	0.88	0.08	< 0.001	0.85	0.07	0.89	0.08	< 0.001
WHtR^a^	0.49	0.06	0.51	0.06	< 0.001	0.52	0.06	0.56	0.06	< 0.001
AVI	14.07	3.28	14.54	3.21	< 0.001	13.42	3.09	15.10	3.25	< 0.001
BAI	26.37	3.46	27.17	3.70	< 0.001	30.03	4.21	32.42	4.73	< 0.001
BRI	3.31	1.10	3.60	1.10	< 0.001	3.78	1.24	4.71	1.37	< 0.001
ABSI	0.076	0.004	0.077	0.005	< 0.001	0.076	0.005	0.080	0.006	< 0.001

*SD* standard deviation, *BMI* body mass index, *WC* waist circumference, *WHR* waist:hip ratio, *WHtR* waist:height ratio, *AVI* abdominal Volume Index, *BAI* body adiposity index, *BRI* body roundness index, *ABSI* a body shape index, *CHD* coronary heart disease

Student’s t-test

^a^Framingham risk score

Table 3 shows the relationship between anthropometric measurements and CHD risk. In an unadjusted logistic regression analysis, the risk of CHD increased as the anthropometric measurements increased, with the exception of BMI in males. In the multivariate-adjusted logistic regression, all these anthropometric measurements were statistically associated with CHD risk in males. After adjusting for all the possible confounders, these anthropometric measurements, except for ABSI, remained as independent indicators of the risk of CHD in females.

**Table 3:** Multivariate-adjusted OR and 95% CI for CHD risk* according to anthropometric parameters: Chinese men and women aged 35 years and over, 2012–2013

	Men (n = 5192)		Women (n = 6055)	
Index	Univariate logestic regression OR (95% Cl)	Multivariate logestic regression OR (95% Cl)	Univariate logestic regression OR (95% Cl)	Multivariate logestic regression OR (95% Cl)
BMI	1.01^b^ (0.99, 1.03)	1.13^a^ (1.10,1.16)	1.08^a^ (1.05, 1.10)	1.12^a^ (1.08,1.16)
WC	1.02^a^ (1.01, 1.02)	1.05^a^ (1.04,1.06)	1.06^a^ (1.05, 1.07)	1.04^a^ (1.02,1.05)
WHR	31.07^a^ (13.65, 70.71)	133.04^a^ (34.88, 507.52)	487.90^a^ (151.25, 1573.89)	10.69^a^ (2.26, 50.50)
WHTR	116.25^a^ (42.54, 317.71)	3.43 × 10^3 a^ (0.60 × 10^3^, 1.97 × 10^4^)	5.03 × 10^4 a^ (1.18 × 10^4^,2.15 × 10^5^)	221.93^a^ (31.28,1574.84)
BAI	1.07^a^ (1.05, 1.08)	1.08^a^ (1.05, 1.11)	1.12^a^ (1.10,1.14)	1.06^a^ (1.03,1.09)
AVI	1.05^a^ (1.03, 1.07)	1.14^a^ (1.10,1.18)	1.17^a^ (1.13,1.20)	1.10^a^ (1.06,1.14)
BRI	1.28^a^ (1.21, 1.34)	1.50^a^ (1.37,1.65)	1.64^a^ (1.54,1.76)	1.27^a^ (1.16,1.39)
ABSI	7.45 × 10^32 a^ (2.22 × 10^27^, 2.50 × 10^38^)	1.13 × 10^15 a^ (1.83 × 10^6^, 7.00 × 10^23^)	3.63 × 10^45 a^ (4.31 × 10^38^, 3.06 × 10^52^)	0.3^b^

*OR* odds ratio, *CI* confidence interval, *BMI* body mass index, *WC* waist circumference, *WHR* waist:hip ratio, *WHtR* waist:height ratio, *ABSI* a body shape index, *AVI* abdominal Volume Index, *BAI* body adiposity index, *BRI* body roundness index, *CHD* coronary heart disease

Adjusted variables included age, hypertension†, diabetes‡, dyslipidaemia§, current smoking, current drinking, education and physical activity

*Medium or high Framingham risk score

†Hypertension was defined as a systolic blood pressure (SBP) ≥140 mmHg, and/or diastolic blood pressure (DBP) ≥90 mmHg, according to the JNC-7 report guidelines

‡Diabetes was diagnosed using the WHO criteria, FPG ≥7 mmol/L (126 mg/dl) and/or being treated for diabetes

§Dyslipidaemia was defined as using lipid-lowering drugs or having one or more of the following measurements: TG ≥1.7 mmol/L, TC ≥5.2 mmol/L, HDL-C < 1.0 mmol/L and LDL-C ≥ 3.4 mmol/L.

^a^*p* < 0.001

^b^*p* > 0.05

Logistic regression analysis

**Fig. 1:**
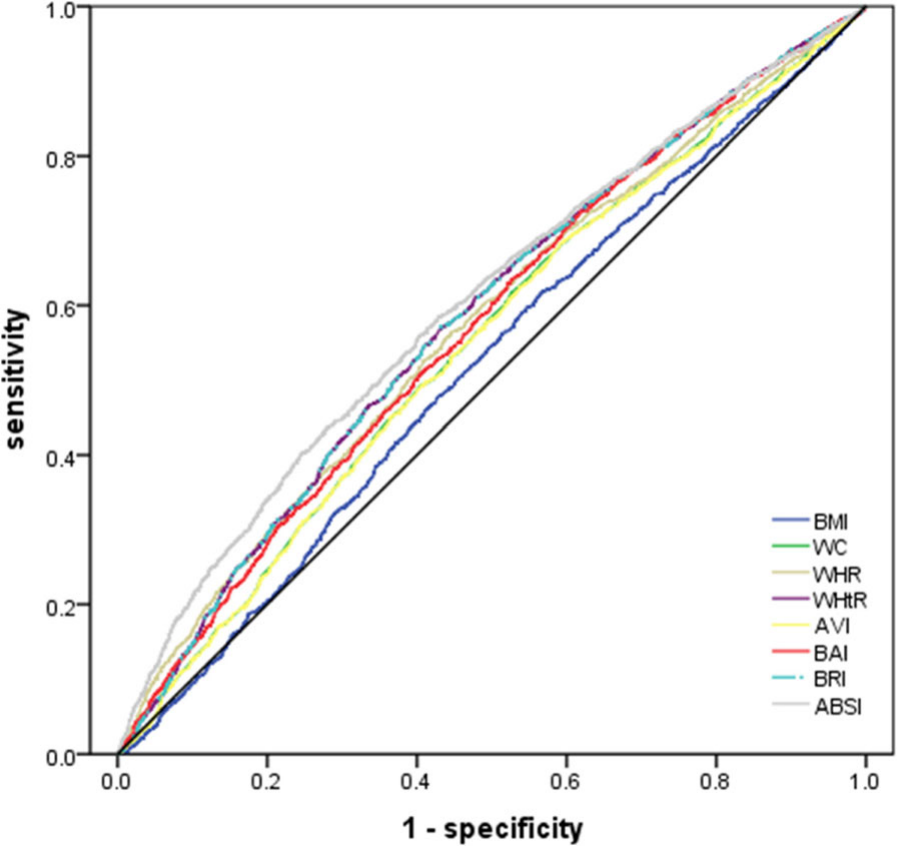
Receiver-operating characteristic curves of anthropometric measurements for the prediction of CHD risk (as expressed by medium or high Framingham risk score) in males: Chinese adults aged 35 years and over, 2012–2013. BMI, Body Mass Index; WC, waist circumference; WHR, waist:hip ratio; WHtR, waist:height ratio; AVI, abdominal Volume Index; BAI, body adiposity index; BRI, body roundness index; ABSI, a body shape index; CHD, coronary heart disease. The areas under the curve for each index were as follows: 0.52 for BMI, 0.55 for WC, 0.57 for WHR, 0.58 for WHtR, 0.55 for AVI, 0.57 for BAI, 0.58 for BRI, and 0.60 for ABSI

**Fig. 2:**
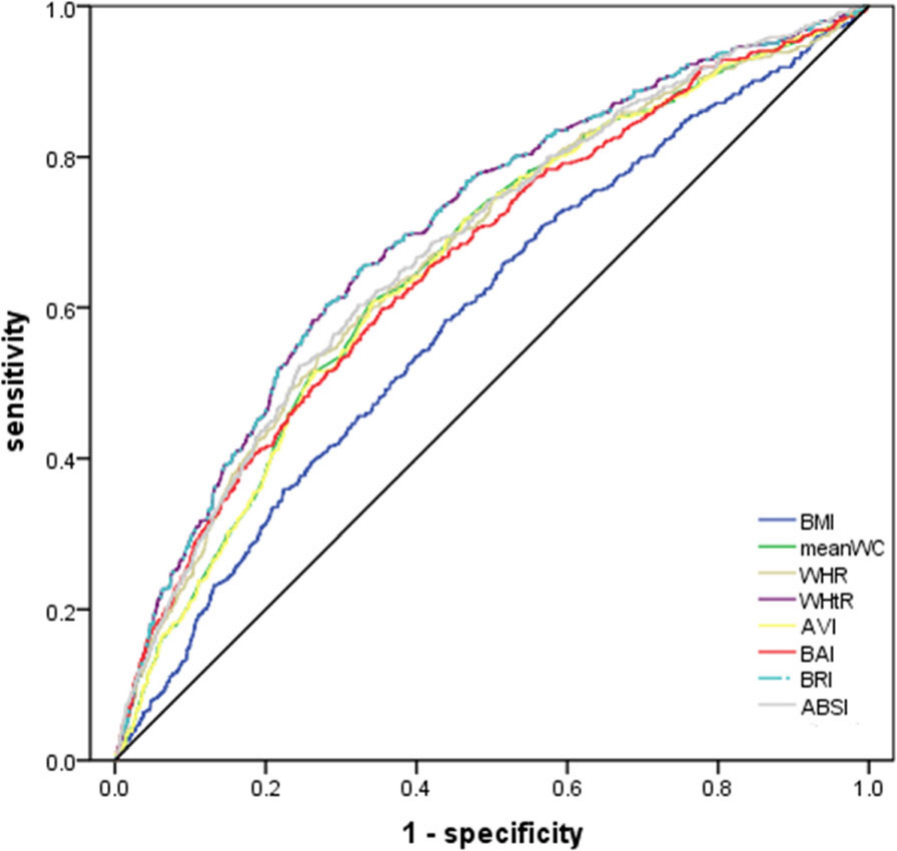
Receiver-operating characteristic curves of anthropometric measurements for the prediction of CHD risk (as expressed by medium or high Framingham risk score) in females: Chinese adults aged 35 years and over, 2012–2013. BMI, Body Mass Index; WC, waist circumference; WHR, waist:hip ratio; WHtR, waist:height ratio; AVI, abdominal Volume Index; BAI, body adiposity index; BRI, body roundness index; ABSI, a body shape index; CHD, coronary heart disease. The areas under the curve for each index were as follows: 0.59 for BMI, 0.66 for WC, 0.67 for WHR, 0.70 for WHtR, 0.66 for AVI, 0.66 for BAI, 0.70 for BRI, and 0.68 for ABSI

Figures 1 and 2 show the most suitable predictors for CHD risk in males and females, respectively. Table 4 provides the AUCs of these obesity parameters. As shown in Table 4 and Fig. 1, ABSI provided the largest AUC value in males, and BMI showed the lowest AUC value, with AUC varying from 0.52 to 0.60. In Fig. 2, WHtR and BRI provided the largest AUC value in females; similarly, BMI showed the lowest AUC value, with AUC varying from 0.59 to 0.70.

**Table 4:** AUC values for anthropometric parameters in estimating CHD risk*: Chinese men and women aged 35 years and over, 2012–2013

	Men		Women	
	AUC	*P*	AUC	*P*
BMI	0.52	< 0.05	0.59	< 0.001
WC	0.55	< 0.001	0.66	< 0.001
WHR	0.57	< 0.001	0.67	< 0.001
WHtR	0.58	< 0.001	0.70	< 0.001
AVI	0.55	< 0.001	0.66	< 0.001
BAI	0.57	< 0.001	0.66	< 0.001
BRI	0.58	< 0.001	0.70	< 0.001
ABSI	0.60	< 0.001	0.68	< 0.001

*AUC* area under the receiver-operating characteristic curve, *BMI* body mass index, *WC* waist circumference, *WHR* waist:hip ratio, *WHtR* waist:height ratio, *AVI* abdominal Volume Index, *BAI* body adiposity index, *BRI* body roundness index, *ABSI* a body shape index, *CHD* coronary heart disease

*Medium or high Framingham risk score

Receiver operating characteristic(ROC) curve analysis

To obtain the optimal cut-off points, the ROC curves and the Youden index were used. The optimal cut-off values of the best indices were WHtR (female: 0.54), BRI (female: 4.21), and ABSI (male: 0.078).

## Discussion

The majority of participants were middle-aged adults from rural areas of China. Compared with females, males were older and had higher blood pressure, WC, height, WHR, and AVI values, as well as significantly higher prevalences of hypertension, previous myocardial infarction, FRS ≥ 10% and smoking. In both males and females, those who had higher CHD risks were more likely to have higher mean anthropometric parameters. In the multivariate-adjusted logistic regression, all these anthropometric measurements were statistically associated with CHD risk in males. After adjusting for all the possible confounders, these anthropometric measurements, except for ABSI, remained as independent indicators of the risk of CHD in females. In males, ABSI had the highest AUC. WHtR and BRI had the highest AUC in females.

Obesity is an excess of body fat [21], and it has become a worldwide epidemic, not only in China but also in other parts of the world. In total, 59% of Chinese are overweight or obesity under the Chinese BMI criteria [22]. The condition is more serious in America, where 70% of adults could be classified as overweight or obese [23]. In our study, 45% of the adults were overweight or obese, according to the WHO criteria, which is lower than the average in China. This difference may be due to the different criteria used.

Obesity is closely related with cardiovascular morbidity and mortality [24]. Similarly, our study showed that almost all anthropometric measurements were associated with CHD risk. Note that BMI was not continually associated with CHD risk in males, likely because BMI cannot differentiate visceral adiposity and overall adiposity, which is more often associated with diseases [25].

Apart from BMI, all the other anthropometric measurements can be used to evaluate the deposition of intra-abdominal fat. In our study, WHtR was the best index for estimating CHD risk in females, which aligned with previous study results [26, 27]. Moreover, we found that BRI and WHtR had nearly the same ability in identify CHD risk, likely because both BRI and WHtR are based on WC and height. Paajanen TA et al. have found that short adults had a higher risk of CHD morbidity and mortality than tall individuals [28]. Henriksson et al. have found that people with short height had a greater risk of suffering from hyperlipidaemia, independent of BMI [29], which may explain why WHtR and BRI could perform better than BMI, WC, and WHR in determining CHD risk in females. WHtR and BRI also performed better than BAI, likely because WC was better than hip circumference in estimating the risk of cardiovascular risk [30].

In our study, ABSI was the best anthropometric index for estimating CHD risk in males. ABSI could avoid the influence of body size on the constituent parts of body [17]. Krakauer et al. have reported that ABSI was positively related with visceral adiposity but negatively related with limb muscle [17]. Limb circumference has been shown to have strong negative correlations with mortality risk [31]. Many studies have shown that ABSI was closely asssociated with some cardiovascular risk factors [14, 15]. However, the relationship between ABSI and CVD remains controversial. Maessen et al. have shown that ABSI was not a suitable index to identify CVD and CVD risk factors in the Netherlands [13]. Similarly, we found that ABSI was not statistically associated with CHD risk in females. Sen et al. have shown that ABSI had some correlation with height and WC [14]. The difference in the predictive ability of ABSI may originate from the Chinese-European and male-female difference in height and WC.

Further, we found that the optimal cut-off value of WHtR in females was 0.54 for CHD risk, which aligned with previous studies. A cross-sectional study conducted by Reci Meseri et al. concluded that 0.55 was the optimal cut-off point of WHtR and that a WHtR value above 0.55 was significantly associated with having medium or high CHD risk in Turkish adults [27]. Moreover, the optimal cut-off value of WHtR in women, which varied from 0.48 to 0.55 [26, 27, 32–34], was still debatable; 0.54 was within this range and close to 0.5 [35], which was more likely to be the optimal cut-off value of WHtR for some cardiometabolic risk factors. It seems that females should maintain a waist circumference that is less than half their height.

Note that the number of females with a medium or high CHD risk was significantly lower than the number of males, likely because the women in our study had a significantly lower prevalence of smoking and lower blood pressure levels. Moreover, previous studies have demonstrated that females had a lower rate of CAD events [36]. Similarly, females in our study had a lower rate of previous myocardial infarction.

### Study limitations

First, the majority of participants were middle-aged adults from rural areas of China, and we could not confirm that the optimal anthropometric indices would perform better than other indices in other subgroups. Second, the cross-sectional study design may be unable to distinguish between cause and effect, and follow-up data are needed.

## Conclusions

Our study showed that the percentage of participants with medium or high CHD risks was significantly higher in males than in females. BMI was not the optimal anthropometric parameter to predict the risk of CHD. ABSI was the best predictor in males, whereas WHtR and BRI were the best indices in females. The optimal cut-off values were as follows: WHtR (females: 0.54), BRI (females: 4.21), ABSI (males: 0.078).

## Additional file

Additional file 1: Health Questionnaire(2012). The questionnaire used in our study was developed for this study. (PDF 124 kb)

## Abbreviations

ABSI: A body shape index
ASCVD: Arteriosclerotic cardiovascular disease
AUC: Area under the receiver-operating characteristic curve
AVI: Abdominal Volume Index
BAI: Body adiposity index
BMI: Body mass index
BRI: Body roundness index
CHD: Coronary heart disease
CI: Confidence interval
FRS: Framingham risk score
HDL: High-density lipoprotein
LDL: Low-density lipoprotein
OR: Odds ratio
SD: Standard deviation
TC: Total cholesterol
TG: Triglycerides
WC: Waist circumference
WHR: Waist:hip ratio
WHtR: Waist:height ratio
