# Supplementary material for: New anthropometric indices or old ones: which perform better in estimating cardiovascular risks in Chinese adults
Source: BMC Cardiovasc Disord. 2018 Jan 30;18:14. doi: 10.1186/s12872-018-0754-z (PMC5789564; doi:10.1186/s12872-018-0754-z)
Supplement: Additional file 1: — Health Questionnaire(2012). The questionnaire used in our study was developed for this study. (PDF 124 kb) [file 12872_2018_754_MOESM1_ESM.pdf]

All information in this questionnaire is confidential.

Health Questionnaire (2012)

Code: □□□ □□□□ **Time**: ________ ________

A. General situation

A1 Name____________ A2 Telephone number

A3 Home address

A4 Gender Male=1 Female=2

A5 Date of birth

A6 Marital status Unmarried=1 Married=2 Remarried=3 Divorced=4 Widowed=5

A7 Educational status Literacy=1 Primary school=2 Junior middle schoo=3 High school=4 Junior College=5 University degree and above=6

A8 Nationality Han=1 Mongolian=2 Korean=3 Hui=4 Manchu=5 other=6

B. Economic status

B1 Annual income Yuan

B2 level of income High=1 Middle=2 Low=3 Poor=4

C. Medical history

		C1 Heart disease	C2 Stroke	C3 Kidney disease	C4 diabetes
(Yes=1, No=2)					
	1				
	2				
	3				---
	4				---
	5				---
	6		---		---
Diagnosis time					

Heart disease: Angina=1 Myocardial infarction=2 Atrial fibrillation=3 Other types of arrhythmia=4 Heart failure=5 other=6

Stroke: Cerebral hemorrhage=1 Cerebral infarction=2 Subarachnoid hemorrhage=3 TIA=4 other=5

Kidney disease: Nephritis=1 Renal insufficiency=2 Kidney stones=3 Kidney tumor=4 Autoimmune disease of the kidney=5 other=6

Diabetes: Type 1 diabetes=1 Type 2 diabetes=2

C5 Have you ever had any other disease **Yes=1( ), No=2**

D.Hypertension related conditions

D1 Are you told by medical staff that you have high blood pressure? **Yes=1, No=2(If "yes", please continue to answer the following questions, if "no", please turn to the next section)**

D2 First diagnosis time Systolic pressure mmHg Diastolic pressure mmHg

D3 Are you taking antihypertensive drugs in the past two weeks? **Yes=1, No=2**

E.Blood lipids and blood sugar

E1 Is there a doctor who tells you that you are suffering from diabetes? Yes=1, No=2**(If "yes", please continue to answer the following questions, if "no", please turn to the next section)**

E2 If you have diabetes, whether you are taking medicine for the last 2 weeks? Yes=1, No=2

E3 Is there a doctor who tells you that you are suffering from dyslipidemia? Yes=1, No=2**(If "yes", please continue to answer the following questions, if "no", please turn to the next section)**

E4 If you have dyslipidemia, whether you are curing it by diet, exercise, or medication for the last 2 weeks? Yes=1，No=2

F.Smoking situation

F1 Have you ever smoked?(at least 1 cigarettes a day, at least 6 months) **Yes=1，No=2(If "yes", please continue to answer the following questions, if "no", please turn to the next section)**

F2 Are you still smoking? **Yes=1，No=2**

F3 How many cigarettes do you smoke on average daily?

F4 Have you ever given up smoking? **Yes=1，No=2**

F5 If you ever quit smoking, how much time do you stop smoking?
<six months=1 More than 6 months=2 More than 1 years=3 More than 2 years=4 Unclear=5

G.Drinking situation

G1 Do you drink regularly?(Drink two or more times a week and last for more than a year)
Yes=1，No=2(If "yes", please continue to answer the following questions, if "no", please turn to the next section)

G2 Are you still drinking? **Yes=1，No=2**

G3 Type, frequency and amount of alcohol drinking G3.1-3.6

Type	Beer	Liquor	wine
Frequency	( )Times / week	( )Times / week	( )Times / week
Amount	( )Bottle / time	( )g / time	( )g / time

G5 Have you ever quit alcohol?**Yes=1，No=2(If "yes", please continue to answer the following questions, if "no", please turn to the next section)**

G6 If you ever quit alcohol, how much time do you stop drinking?
<six months=1 More than 6 months=2 More than 1 years=3 More than 2 years=4 Unclear=5

H. life style

H1 Do you exercise regularly? **Yes=1，No=2(If "yes", please continue to answer the following questions, if "no", please turn to the next section)**

H2 The average time and frequency of your weekly exercise

H3 What is your most common exercise style?
Walking =1 running =2 swimming =3 ball sports =4 Qigong =5 mountaineering =6 other =7

H4 Did you take any medicine in the last 2 weeks? Yes =1 No =2
If so, what kind of drugs are taken: ____________________

At the end of the investigation, please check if there are any omissions and mistakes.

investigators signature: (required *)

the survey time: date (required *)
